# Supplementary figures and images for: Establishment and application of unbiased in vitro drug screening assays for the identification of compounds against Echinococcus granulosus sensu stricto
Source: PLoS Negl Trop Dis. 2023 Aug 4;17(8):e0011343. doi: 10.1371/journal.pntd.0011343 (PMC10431624; doi:10.1371/journal.pntd.0011343)

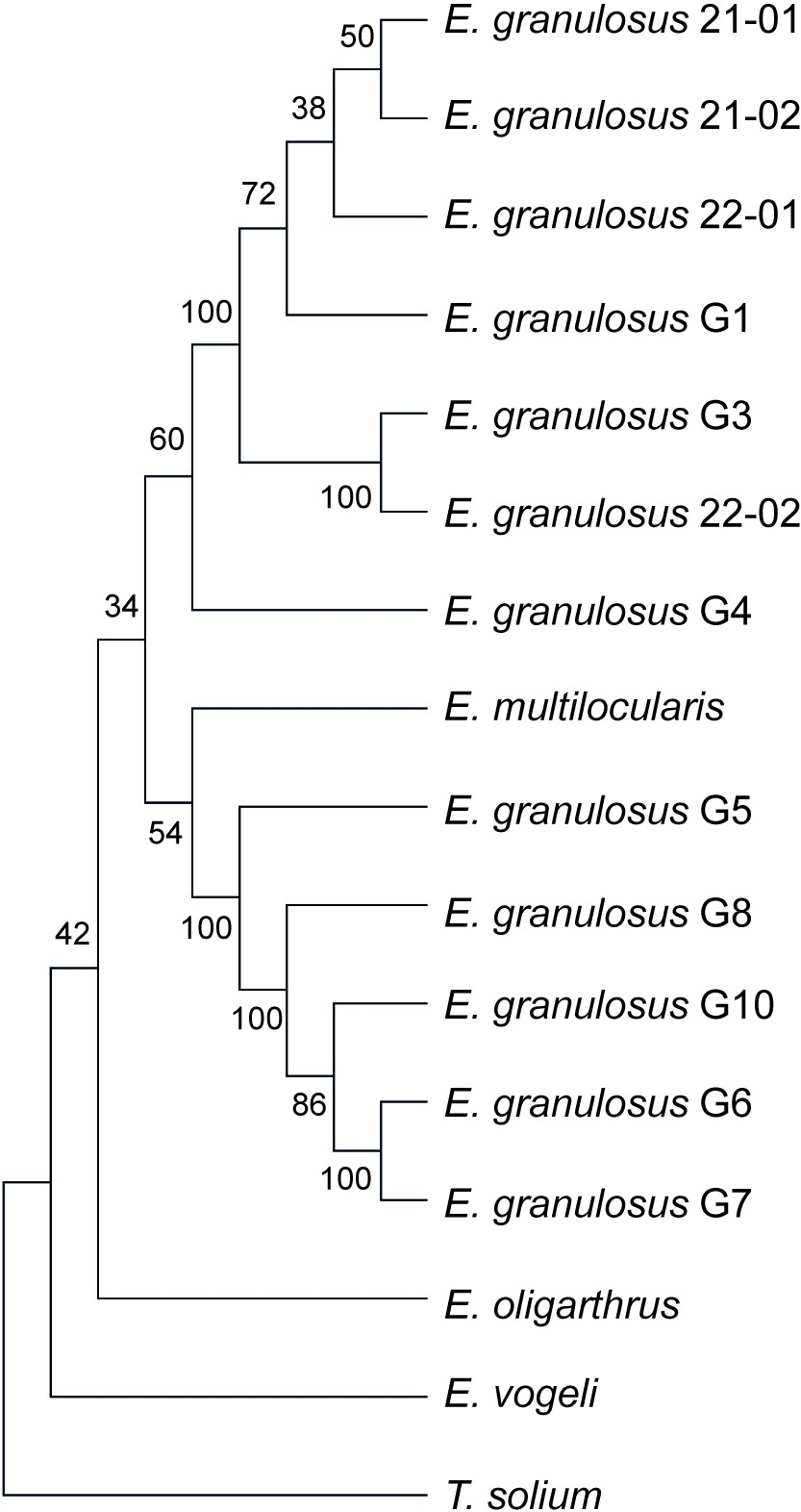

Supplement: S1 Fig — Four isolates of E. granulosus s.s. were genotyped using a concatenated sequence of the four mitochondrial markers atp 6, nad I, cox I and rrnL. The tree was generated according to the maximum likelihood method with the HKY+G+I model. (TIF) [file pntd.0011343.s001.tif]
